# Performance evaluation of grid connected photovoltaic pilot plant in saharan climate using experimental and numerical analysis

**DOI:** 10.1038/s41598-025-19300-3

**Published:** 2025-09-12

**Authors:** Tarek Hazem, Hocine Cheghib, Amor Fezzani, Nabil Kahoul, Mohamed Rafik Sari, Farhan Lafta Rashid, Arman Ameen, Mohamed Kezzar, Ibrahim Mahariq

**Affiliations:** 1 Laboratoire des Systèmes Electromécaniques, (LSELM), Badji Mokhtar-Annaba University, 12, P.O.Box.23000, Annaba, Algeria; 2 Unité de Recherche Appliquée en Energies Renouvelables (URAER), Centre de Développement des Energies Renouvelables (CDER), 47133 Ghardaia, Algeria; 3 Mechanics of Materials and Plant Maintenance Research Laboratory (LR3MI), Mechanical Engineering Department, Faculty of Engineering, Badji Mokhtar University of Annaba (UBMA), PO Box 12, 23052 Annaba, Algeria; 4 Petroleum Engineering Department, College of Engineering, University of Kerbala, Karbala, 5600 Iraq; 5 Department of Building Engineering, Energy Systems and Sustainability Science, University of Gävle, 801 76 Gävle, Sweden; 6 Materials and Energy Engineering Laboratory (LMGE), Technology Department, Faculty of Technology, 20 Aout 1955 University of Skikda, PO Box 26, 21000 Skikda, Algeria; 7 Department of Mathematics, Saveetha School of Engineering, Saveetha Institute of Medical and Technical Sciences, Saveetha University, Chennai, Tamil Nadu 602105 India; 8 Najjad Zeenni Faculty of Engineering, Al Quds University, Jerusalem, Palestine; 9 University College, Korea University, Seoul, 02481 South Korea; 10 Department of Medical Research, China Medical University Hospital, China Medical University, Taichung, Taiwan; 11 Applied Science Research Center, Applied Science Private University, Amman, Jordan

**Keywords:** Photovoltaic system performance, Performance ratio (PR), PVGIS simulation, Desert climate PV analysis, Grid-connected solar plant, Photovoltaics, Solar cells

## Abstract

Algeria has made significant progress in developing its renewable energy capacity, particularly in utilizing solar resources to meet growing electricity demand. As part of this effort, several large-scale photovoltaic (PV) plants have been established in the southern desert regions. This study evaluates the performance of a 1.1 MW grid-connected pilot PV plant located in Ghardaia, consisting of eight subfields, six fixed and two equipped with motorized solar tracking, utilizing multiple PV technologies. The plant’s performance is assessed over a 12-month period (January–December 2016) using both experimental data and simulations conducted with the PVGIS tool. Key performance indicators include the capacity factor (CF), reference yield (Yr), final yield (Yf), and performance ratio (PR). Experimentally measured annual averages were CF = 22.66%, Yr = 195.79 kWh/kWp, Yf = 154.96 kWh/kWp, and PR = 0.80. PVGIS simulations returned CF = 23.66%, Yr = 205.76 kWh/kWp, Yf = 173.83 kWh/kWp, and PR = 0.85. Strong correlations were found between PR and air temperature (R² = 0.8995), output power and irradiation (R² = 0.8577), and output power and module temperature (R² = 0.8577). These results confirm the plant’s strong performance and support its scalability across Algeria’s harsh Saharan climate.

## Introduction

The transition to sustainable energy is essential for addressing two critical global challenges; dependence on fossil fuels and the accelerating threat of climate change^1^. Photovoltaic (PV) systems are among the most promising renewable solutions, as they convert sunlight into electricity, providing power for residential, commercial, and community applications^2^. These systems are not only emission-free but also operate quietly and reliably, making them an environmentally friendly energy source^3^. A nation’s electricity usage is a major indicator of its development^4^. This is evident in the exponential growth of the global PV market, with installations rapidly increasing^5^. By 2022, global PV capacity had surpassed 1.185 GW and is projected to continue growing in the years to come^6^. Several factors contribute to this widespread adoption, including the low technical complexity of PV systems, their demonstrated economic viability, and increasing awareness of their ecological benefits^7^. Algeria, located in the “Sunbelt” region, receives an average annual solar radiation of approximately 2000 kWh/m^2^, among the highest in the world, making it ideal for solar energy generation, particularly in the southern and high plateau regions^8^. However, the harsh climate, especially in the south, presents major challenges for PV deployment. Extreme temperatures, often exceeding 50 °C (122 °F), can significantly reduce panel efficiency and reliability^9^. Additionally, abrasive sand and dust storms can damage panel surfaces, reducing their lifespan and increasing maintenance demands^10^. Limited rainfall and water availability hinder conventional cooling methods, which can result in overheating and further efficiency losses^11^. Moreover, sandstorms and high temperatures accelerate degradation of PV system components, such as cables and inverters^12^. Since 2011, Algeria has increasingly integrated environmental considerations into its economic policies, promoting clean energy through a range of national renewable energy programs^13^. In 2015, these programs were updated to place greater emphasis on solar energy^2^. With domestic electricity demand projected to reach 150 TWh and gas consumption expected to hit 55 billion m^3^ by 2030, Algeria’s ambitious energy plan aims to reduce reliance on fossil fuels, particularly natural gas, and achieve 40% renewable energy capacity by 2030^14^. The country’s favorable geographic position offers significant potential to become a key player in the global solar energy sector^15^. Although the national renewable energy program set a goal of reaching 22 GW by 2030, various challenges have delayed its implementation and influenced national energy policy^16^. In 2020, Algeria’s Prime Minister announced a transformative plan to generate 15,000 MW of clean electricity by 2035, with a target of 4,000 MW by 2024, marking a significant milestone in the country’s transition to green energy. Currently, Algeria hosts 25 grid-connected renewable energy plants, including one wind plant, producing a combined total of approximately 354.3 MW, located across both the southern and northern regions^17^. Given this progress, there is an increasing need for experimental evaluations of system performance, validation through PV simulation software, and analyses of environmental and operational factors affecting energy output, to ensure technical viability and reliability^18^. In recent years, numerous studies have been conducted to assess the performance of grid-connected PV plants across North Africa, the Sahel, the Middle East, and other regions with diverse climatic conditions^19^. For example, in 2022, researchers analyzed three silicon-based grid-connected PV systems located in El Jadida, Morocco^20^, their findings indicated that monocrystalline and polycrystalline silicon systems outperformed micro-morph tandem technology under Mediterranean conditions, the monocrystalline and polycrystalline systems yielded an average final output of 4.98 kWh/kWp/day, a performance ratio (PR) of 80.73%, and a capacity factor (CF) of 20.76%, while the micromorph tandem system recorded values of 4.65 kWh/kWp/day, 75.15% PR, and 19.38% CF, respectively. Similarly, in 2023, Sami Florent Palm et al.^21^ analyzed Burkina Faso’s largest grid-connected PV system (33 MW) over the period 2019–2021. Their results showed that rainy months with high irradiation produced the highest energy output, while efficiency declined during hotter seasons due to elevated module temperatures. Over the study period, the system’s efficiency slightly declined from 12.29% to 12.10%, with the PR ranging from 80.73% to 79.36% and the CF between 19.89% and 19.33%. Earlier, in 2019, researchers evaluated a 20.4 kW grid-connected PV system installed in 2014 in Muscat, Oman^22^. Key performance metrics included a daily reference yield of 5.59 kWh/kWp/day, array yield of 3.78 kWh/kWp/day, final yield of 3.64 kWh/kWp/day, PR of 67%, CF of 15%, and system efficiency of 10.3%. Daily losses included capture losses (1.82 h/day), system losses (0.14 h/day), and temperature-related losses (2.95 h/day). Although soiling was noted to reduce energy output, its exact impact was not quantified. In 2022, a study investigated the technical, economic, and environmental aspects of a 425-kW building-applied PV (BAPV) system in Saudi Arabia^23^. The system performed well, achieving an annual PR of 78.09%. More recently, in 2024, researchers assessed shading effects on PV systems. In Indonesia using PVsyst software, evaluating four designs with varying tilt and azimuth angles^24^ PR values ranged between 0.815 and 0.817, while energy losses varied from 11.04 MWh to 11.67 MWh. Another study conducted in 2021 examined a 7.8 kWp rooftop PV system. In Malaysia over two years, utilizing PVsyst for performance analysis and HOMER-Pro for economic assessment^25^. In 2019, the system achieved an optimal PR of 75.72%, average daily losses of 1.68 kWh/kWp, a CF of 13–16%, and an efficiency range of 10–12%. A tilt angle of 5° increased annual energy output by 4.8%. In France, a 2018 study of a 2.4 kWp grid-connected PV system (2018–2020) revealed annual energy fluctuations, peaking in 2019 at 3,246.47 kWh. The study compared PVGIS, PVWatts, and HOMER, noting that PVGIS tended to overestimate, while the other two slightly underestimated performance. In Algeria’s arid desert climate, researchers have also examined the performance of photovoltaic plants^26^. For instance, in Ethiopia, an experimental study evaluated a 10 kWp solar system with battery storage. The analysis highlighted underutilized batteries and excessive grid reliance, leading the researchers to suggest optimizing the system using real-time data and simulations. They proposed a smaller 5 kWp system as a more efficient and economically viable alternative^27^. In 2021, researchers analyzed the performance of a 23.92 MWp solar PV plant in El Bayadh, Algeria, over 36 months (March 2017–February 2020)^28^. Key metrics such as PR, yield factor, and capacity utilization factor were evaluated, revealing strong alignment between actual output and PVsyst/SolarGIS model predictions. A high correlation was found between module temperature and PR (91%), with an annual PR degradation rate of 0.76%. In another study, a large-scale grid-connected PV plant (LS-PVPP) in Adrar, Algeria, was assessed using north-tabo modeling and field data^29^. The results highlighted a final yield of 4.98 kWh/kWp/day, a PR of 71.67%, and a CF of 20.72%. The authors emphasized the plant’s potential for carbon emissions reduction, achievement of target PR, and further energy generation and revenue potential. Several studies have examined PV performance in arid climates^30^, focusing on energy yield, degradation, and temperature effects. However, few studies have investigated the comparative performance of fixed and tracking PV systems using both simulated and empirical data, especially under the extreme conditions of the Saharan environment. Moreover, no comparative analysis using both PVGIS simulations and empirical measurements for fixed and tracking systems under Saharan conditions has been conducted. This gap limits our ability to accurately assess the real-world performance and reliability of different PV technologies in harsh Saharan conditions, which is essential for optimizing system design, investment decisions, and operational strategies for large-scale solar deployment in these regions. Therefore, this study aims to evaluate the performance of a 1.1 MW grid-connected pilot PV plant located in Ghardaïa, Algeria, by comparing simulated energy outputs from PVGIS with empirical measurements under Saharan climatic conditions, considering both fixed and tracking PV systems to provide a comprehensive analysis of key performance indicators essential for future solar energy deployment in the region.

The main contributions of this study are as follows:

1. Providing a comparative analysis between simulated and measured PV performance under Saharan conditions,
2. Evaluating the impact of using tracking systems versus fixed installations on energy yield and performance ratios, and.
3. Offering practical insights for optimizing PV system design in harsh arid climates to support the large-scale deployment of solar energy in Algeria.

This study conducts a comparative evaluation of a grid-connected photovoltaic pilot plant using both experimental data and the PVGIS software simulator. The goal is to assess the plant’s performance to support its adoption and promote the broader implementation of this energy generation method across Algeria’s desert climate. The paper is structured as follows. Section 2 describes the geographical location, data acquisition process, and hardware configuration of the PV pilot plant. Section 3 presents the methodology for technical performance analysis, including definitions and descriptions of key performance parameters. Section 4 reports the results of the performance evaluation, offering a comparative analysis of the simulated and measured energy output as well as performance metrics. Finally, Sect. 5 concludes the study by summarizing key findings and outlining future research directions.

## Material

### Description of the composed structure system 1.1 mwp

#### Geographical location of the PV plant

**Fig. 1:**
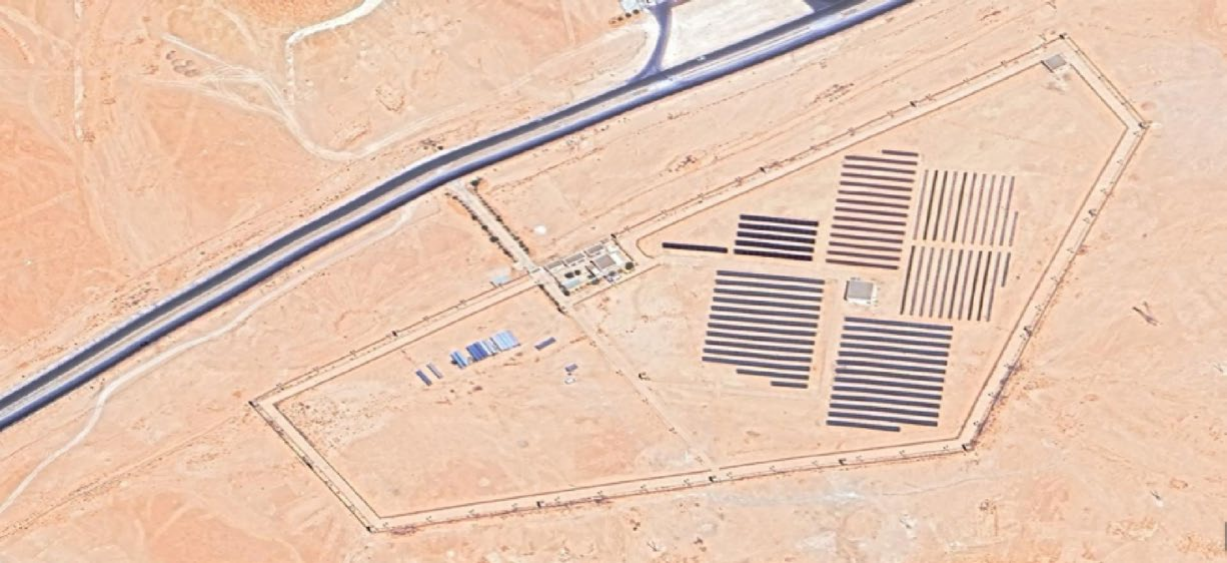
Global view of subfields of (1.1MW_p_) PV plant, Oued Nechou, Ghardaia, Algeria.

The pilot plant (Oued Nechou) is located in the southern region of Algeria (32°34′43″N, 3°41′55″E), near Ghardaia (see Fig. 1). This region experiences a harsh, hot, and arid climate throughout the year, receiving over 3,000 h of sunshine annually. The horizontal global solar radiation in this area exceeds 6,000 Wh/m^2^ per year^31^. The climate is extreme, with clearly defined summer and winter seasons. The hot season extends from June 6 to September 11, with average daily high temperatures above 35 °C. July is the hottest month, with average highs of 40 °C and average lows of 27.8 °C. In contrast, the cool season spans from November 17 to March 6, during which average temperatures remain below 20.6 °C. January is the coldest month, with average lows of 6.7 °C and highs of 16.1 °C. The transition out of the cold season is often accompanied by heavy dust storms, while summer temperatures can exceed 50 °C^29^. Figure 1 illustrates the geographical location of the Oued Nechou PV pilot plant in the Ghardaia region of Algeria.

The map in Fig. 1 was generated using PVGIS 5.2 (European Commission, Joint Research Centre) with the PVGIS-SARAH database, accessed via : https://re.jrc.ec.europa.eu/pvg_tools/en/.

### Technical specifications

Table 1 shows detailed specifications of the eight sub-fields.

**Table 1:** Descriptions of the eight sub-fields.

Subfield	Field power (kWp)	Structure type	PV technology
1	105	Motorized	Monorystalline silicon (mono-Si)
2	98.5	Motorized	Polycrystalline silicon (poly-Si)
3	100.80	Fixed	Thin layers (Cd-Te)
4	100.11	Fixed	Amorphous silicon (a-Si)
5	105.00	Fixed	Monocrystaline silicon
6	98.70	Fixed	Polycrystalline silicon
7	255.00	Fixed	Monocrystaline silicon
8	258.50	Fixed	Polycrystalline silicon

**Fig. 2:**
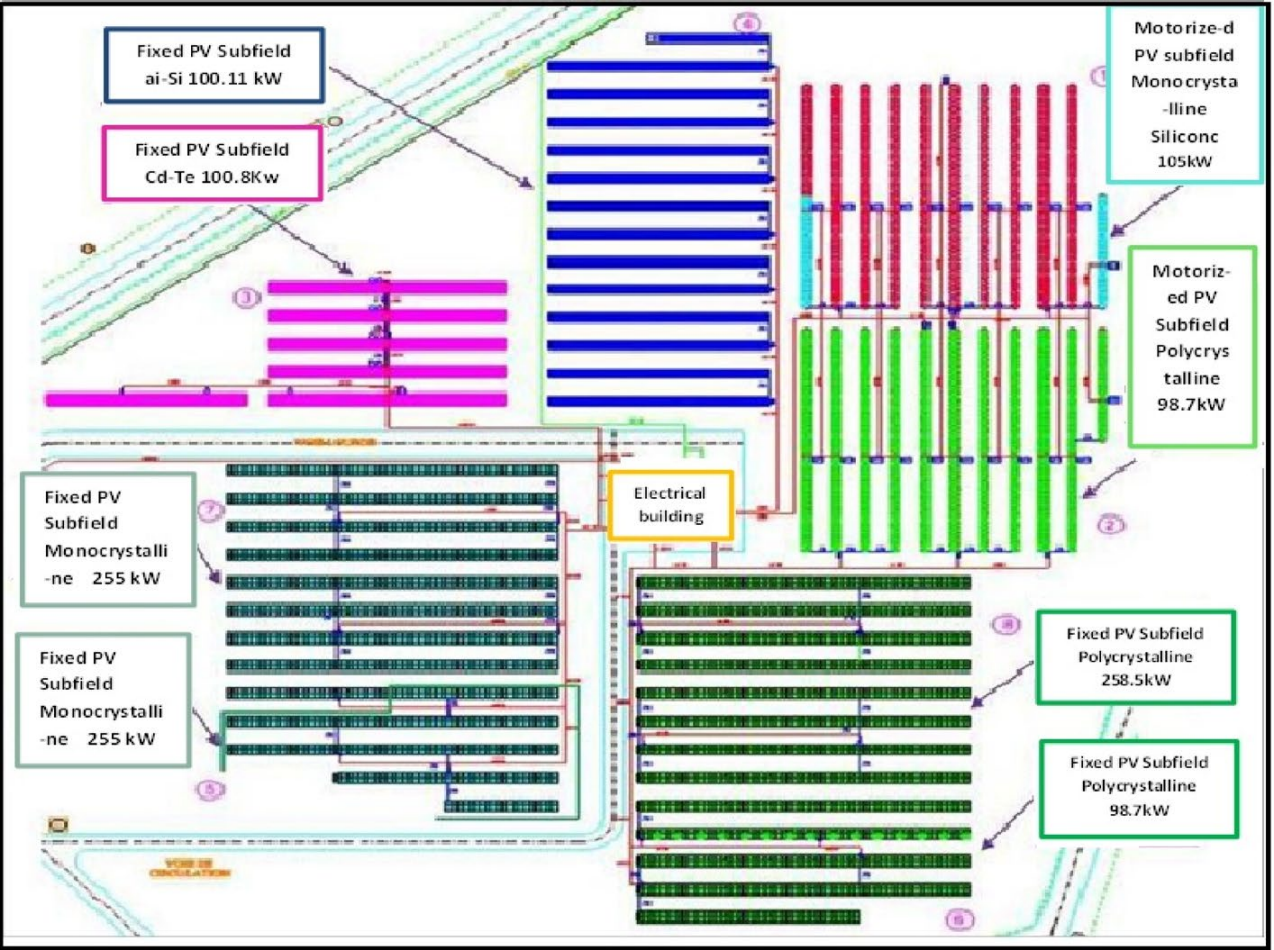
Scheme of 8 subfields distribution of the PV pilot plant Oued Nechou.

The grid-integrated PV pilot plant comprises several key components. Solar panels capture and convert sunlight into direct current (DC) electricity, which is subsequently transformed into alternating current (AC) by inverters before being fed into the grid. The system also includes a robust mounting structure, secure cabling, and various electrical accessories to ensure optimal performance and safety. Figure 2 illustrates the schematic layout, highlighting the essential components:

- Solar panels are arranged in series and parallel configurations, depending on the size of the PV array, to maximize the conversion of incident solar radiation into DC power.
- Maximum Power Point Tracking (MPPT) technology dynamically tracks the optimal operating point of each solar module, ensuring maximum DC power generation under varying sunlight conditions^32^.
- The grid-connected DC/AC inverter plays a critical role in converting DC power into grid-compliant AC electricity, enabling safe and synchronized integration with the utility grid^33^.
- Grid connection requires adherence to rigorous safety standards. DC/AC breakers, fuses, and other protective devices are implemented in compliance with local utility regulations to ensure a secure and reliable connection.
- Dual-axis solar trackers enhance energy yield; motorized systems in selected subfields dynamically follow the sun’s trajectory, maximizing solar irradiance capture^34^.

Table 2 presents the four distinct types of solar panels used in the system, detailing their technical specifications, including nominal inverter (DC/AC) power and panel brand.

**Table 2:** Specifications of PV panel technologies used at the (1.1MW_p_) pilot plant.

Nominal values	Poly-Si	Cd-Te	a-Si	Mono-Si
Maximum power (+/−5%) PM (W)	240	80	103	250
Voltage at MPP. V(V)	29.21	71.2	17.4	30.35
Current at MPP. I (A)	8.21	1.12	5.57	8.24
Open circuit voltage Voc(V)	37.16	91.5	23.7	37.62
Short circuit current Isc(A)	8.73	1.22	6.72	8.79
Temperature coefficient of PMpp (%°C)	−0.43	−0.25	−0.2	−0.43
Temperature coefficient of Voc (%C) (˃25°c)	−0.32	−0.25	−0.33	−0.34
Temperature coefficient of Voc (%°C) 40°c to25°)	−0.32	−0.20	−0.33	−0.34
(Isc) Temperature coefficient of Isc (%°C)	+ 0.04	+ 0.04	+ 0.08	+ 0.03
Module efficiencyof Isc. Η (%)	+ 0.04	11.11	6.9	+ 0.03
Inverter (DC/AC) nominal power: 96 kW European, efficiency: 97.17%.				
Pnel Marque	ATERSA	FIRST SOLAR	SCHOTT PROTECT	ATERSA

## Methodology

### System configuration (1.1MW_p_ pilot plant)

In the PV pilot plant project, there are eight different PV subfields utilizing various technologies. Six of the subfields are fixed systems with a combined maximum power output of 918.00 kWp, while the remaining two are equipped with motorized tracking systems, contributing a maximum power output of 203.5 kWp. The layout and distribution of the PV plant’s subfields are clearly illustrated in Fig. 2.

### Performances analysis of the solar photovoltaic plants (SPVP)

**Fig. 3:**
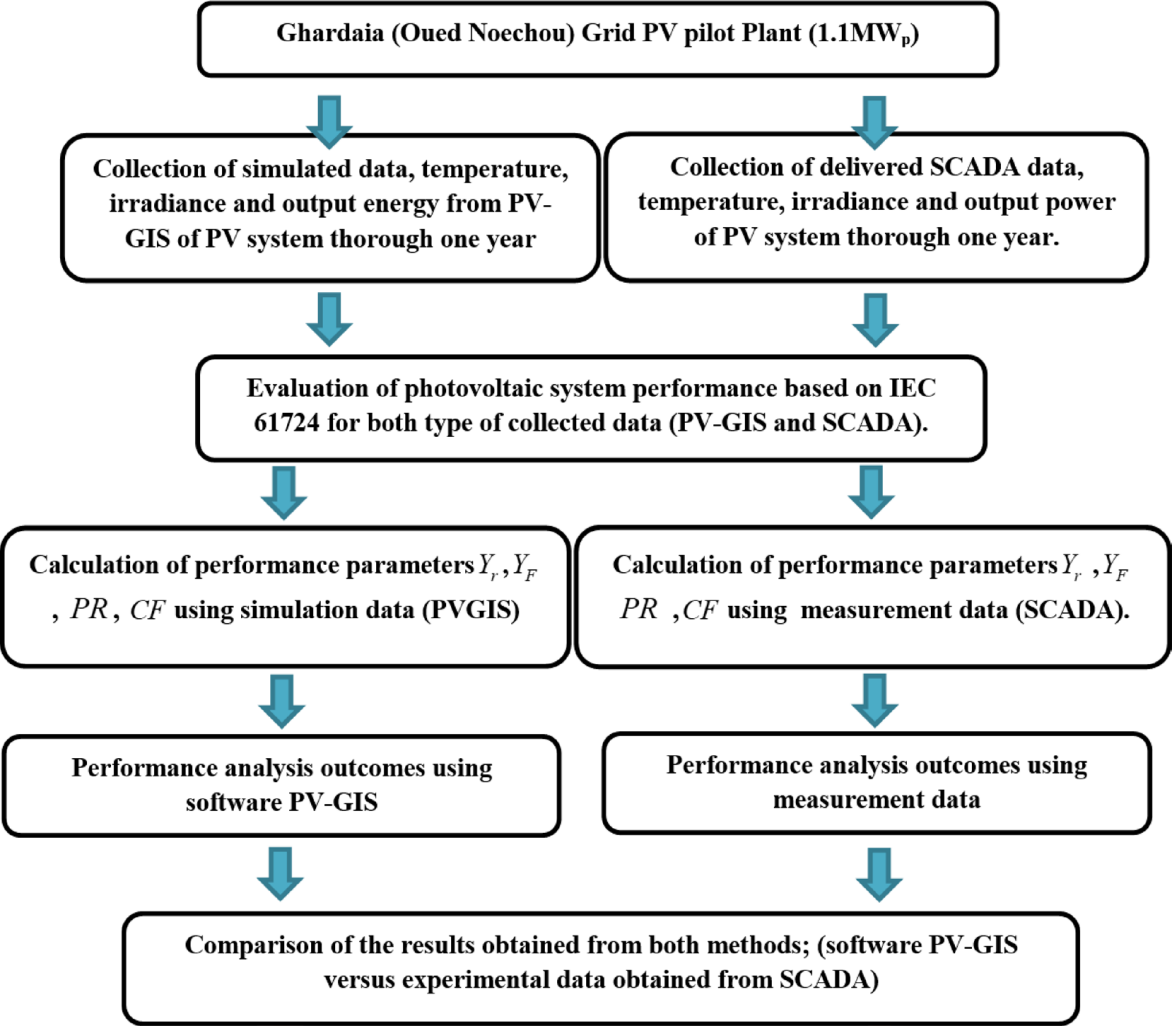
Methodology employed in this study.

Technical indices obtained from on-site data collection were used to analyze the performance of the SPVP pilot plant. The evaluation was based on performance indicators established by the International Energy Agency’s Photovoltaic Power Systems Program, in accordance with the IEC 61,724 standard^15^. Data were recorded every 30 min using a SCADA-based monitoring system, strictly adhering to the performance evaluation guidelines outlined in IEC 61,724^35^. Real-time monitoring of key variables, such as solar irradiance, ambient temperature, and power generation, was implemented. The overall methodology is illustrated in Fig. 3, and the following subsections provide definitions of the key performance indices used to evaluate the solar PV pilot plant.

### Solar radiation and temperature relationship

Solar radiation and temperature are closely interconnected variables that significantly influence the performance of PV systems^36^. Understanding the interaction between energy output and system conditions is essential for developing effective strategies to maximize power generation. The total power output of a PV system is sensitive to the combined effects of solar radiation and temperature. While increased solar radiation typically enhances energy production, this benefit is often offset by elevated panel temperatures, which reduces efficiency. As temperatures rise, solar panels convert less sunlight into electricity^7^. Therefore, the dynamic interplay between solar radiation and temperature is a critical factor in determining the maximum achievable energy yield from a PV system^37^.

The total radiation is expressed as:

1 $${\text{H}}_{\text{t}}={\uptau\:}{\sum\:}_{\text{i}=1}^{\text{N}}{\text{G}}_{\text{m}\text{e}\text{s},\text{i}}$$

Where:

$\text{H}$ is the solar radiation, expressed in (kWh/ m²).

$\:\tau\:$ is a sampling time or recording interval;$\:{\text{G}}_{\text{m}\text{e}\text{s},\text{i}}$ is the measured value of the in-plane irradiance at interval “i” and N is the number of intervals along the entire duration of measurements TM (N = TM/$\tau$).

Module temperature is a function of both ambient temperature and the level of irradiance incident on the module surface. In regions with high solar irradiance, the majority of solar energy production occurs when the module operating temperature significantly exceeds 25 °C. The temperature coefficient expresses the instantaneous rate of change in power output as a function of the module’s operating temperature. Module temperature is primarily influenced by ambient air temperature and solar irradiance^38^.

2 $$\:{\text{T}}_{\text{c}}={\text{T}}_{\text{a}}+\frac{{\text{G}}_{\text{i}\text{m}\text{e}\text{a}\text{s}}}{{\text{G}}_{\text{i}\text{N}\text{O}\text{C}\text{T}}}({\text{T}}_{\text{N}\text{O}\text{C}\text{T}}-20)$$

Where:

G measured value of the in-plane irradiance at the particular time.

NOCT nominal temperature operation cell.

T_c_ module temperature.

T_a_ ambient temperature.

G_i, NOCT_ 800 W/m^2^.

T_NOCT_=43 °C.

#### Energy output

Energy (E_AC_) denotes the AC electrical energy generated by a PV plant. It quantifies the total AC power output produced by the PV system, which can either be fed into the electrical grid or consumed on-site. This energy is commonly measured in kilowatt-hours (kWh) over defined time intervals, such as a day, month, or year^39^.

The total daily (E_AC, d),_ monthly (E_AC, m_), or yearly (E_AC, y_) energy generated by the PV Plant (kWh)^15^. A solar PV plant’s cumulative energy output over time is defined as follows:

Energy (E_AC_) refers to the AC electrical energy generated by a PV plant. It represents the total AC power output produced by the PV system, which can either be fed into the electrical grid or used on-site. This energy is typically measured in kWh over defined time intervals such as daily, monthly, or annually^25^. The total energy generated by the PV plant over a specific period is denoted as E_AC, d_​ for daily energy, E_AC, m_​ for monthly energy, and E_AC, y_ for yearly energy. The cumulative output energy of a solar PV plant over time is calculated using these parameters.

3 $${\text{E}}_{{{\text{AC}}}} = \tau \sum\nolimits_{{i = 1}}^{N} {{\text{P}}_{{{\text{mes}},i}} }$$

Where E_AC_ is expressed in (kWh); $\:{\text{P}}_{\text{m}\text{e}\text{s},\text{i}}$ is expressed in (kW). The output power value of the panel under NOCT test is always less than the output power of the same panel under standard test conditions (STC)^38^.

##### System yields:

These include array yield, final yield, and reference yield, which measure the actual PV system performance relative to its maximum capacity. System yield can be evaluated across different time periods, daily, monthly, or annually, to analyze both short-term and long-term performance trends^39^.

##### Final yield (Y_f_):

Final yield is a performance metric for solar PV systems, representing the ratio of the system’s AC energy output to the peak power of the installed PV array. It is measured in (kWh/kW) and can be computed over various time intervals, daily, monthly, or yearly. When calculated over a year, it is referred to as the annual yield^40^.

4 $$\:{\text{Y}}_{\text{f}}=\frac{{\text{E}}_{\text{A}\text{C}}}{{\text{P}}_{\text{P}\text{V}}}$$

Where:

$\:{\text{E}}_{\text{A}\text{C}}$ : Generated energy (kWh).

$\:{\text{P}}_{\text{P}\text{V}}$ : Peak power of the pilot plant (kW).

##### Reference yield ( Y_r_):

The reference yield (Y_*r*_) of a PV plant is a measure indicating the equivalent hours of sunlight at STC. It is derived by dividing the total in-plane solar radiation (H_*t*_) incident on the plant by the STC reference irradiance (H_R_), which is 1 kW/m^2^. This metric captures the solar energy availability at the plant’s specific location, accounting for factors such as orientation and tilt angle^41^. The mathematical expression for Y_r_ is given in Eq. (5)^42^.

5 $$\:{\text{Y}}_{\text{r}}=\frac{{\text{H}}_{\text{t}\:\:}\:(\text{k}\text{W}\text{h}/{\text{m}}^{2})}{1\:(\text{k}\text{W}/{\text{m}}^{2})}$$

##### Performance ratio:

The performance ratio (PR) is a key parameter used to evaluate the efficiency of a PV power plant. It is defined as the ratio of the plant’s actual electrical energy output to its theoretical electrical energy output^43^. Unlike other parameters, the PR is independent of the plant’s geographic location or panel orientation.

The theoretical output represents the maximum possible energy that the plant could produce under ideal conditions^44^, whereas the actual output reflects the energy generated under real-world operating conditions influenced by various factors^35^.

One critical factor affecting the PR is temperature; higher temperatures typically reduce the PR. This is because the efficiency of solar cells decreases as temperature increases, thereby reducing their ability to convert sunlight into electricity^38^. Another important factor is irradiance, which directly impacts PV system output^17^. Although higher irradiance generally boosts energy production and may enhance the PR, excessive irradiance can sometimes cause efficiency losses in certain PV systems^7^.

The mathematical formula used to calculate PR is as follows^15^:

6 $$\:\text{P}\text{R}=\frac{{\text{Y}}_{\text{f}}}{{\text{Y}}_{\text{r}}}\:=\:\frac{\text{R}\text{e}\text{a}\text{l}\:\text{p}\text{r}\text{o}\text{d}\text{u}\text{c}\text{t}\text{i}\text{o}\text{n}\left(\text{k}\text{W}\text{h}\right)}{\text{T}\text{h}\text{e}\text{o}\text{r}\text{i}\text{c}\text{a}\text{l}\:\text{p}\text{r}\text{o}\text{d}\text{u}\text{c}\text{t}\text{i}\text{o}\text{n}\left(\text{k}\text{w}\text{h}\right)}$$

##### Capacity factor:

Capacity Factor (CF) for a solar PV plant is the ratio of actual electrical output of the plant over a given period of time to the capacity that its maximum power output could have achieved had it operated continuously at 100% capacity. This same metric has the use of comparing the real-world electricity generation against its theoretical best that is able to be reached under STC^33^ to analyze the efficiency of the plant in operation. It is represented in percentages and it is computed as below:

$$\:\:\text{C}\text{F}=\frac{\text{T}\text{o}\text{t}\text{a}\text{l}\:\text{a}\text{c}\text{t}\text{u}\text{a}\text{l}\:\text{e}\text{l}\text{e}\text{c}\text{t}\text{r}\text{i}\text{c}\text{i}\text{t}\text{y}\:\text{g}\text{e}\text{n}\text{e}\text{r}\text{a}\text{t}\text{e}\text{d}}{\text{M}\text{a}\text{x}\text{i}\text{m}\text{u}\text{m}\:\text{p}\text{o}\text{s}\text{s}\text{i}\text{b}\text{l}\text{e}\:\text{e}\text{l}\text{e}\text{c}\text{t}\text{r}\text{i}\text{c}\text{i}\text{t}\text{y}\:\text{g}\text{e}\text{n}\text{e}\text{r}\text{a}\text{t}\text{i}\text{o}\text{n}}\text{*}100\text{\%}$$

7 $$\:\text{C}\text{F}=\frac{{\text{Y}}_{\text{f}}}{24\text{*}\text{n}\text{d}}=\frac{{\text{E}}_{\text{A}\text{C},\text{T}}}{{\text{P}}_{\text{P}\text{V},\text{r}\text{a}\text{t}\text{e}\text{d}}\text{*}8760}\text{*}100\text{\%}$$

### Simulation software

Simulation software for PV systems is essential for various aspects of solar energy planning, design^15^, and optimization. These programs take into account various factors such as:

- Location (latitude, longitude, altitude).
- Solar resource data (irradiance, sunshine hours).
- System size and configuration (number and type of panels, inverter capacity).
- Tilt angle and orientation of the panels.
- Shading from buildings or trees.
- System losses (electrical resistance, inverter efficiency…etc.)

There are several simulation software tools available for designing, analyzing, and simulating PV systems^26^; such as:

- PV-SOL: Photovoltaic Solar or Solution.
- PV-SYST: Photovoltaic System software.
- PV-GIS: Photovoltaic Geographical Information System.
- HOMER-PRO: Hybrid Optimization of Multiple Energy Resources.
- SAM: System Advisor Model.
- RETScreen: Renewable Energy Technology Screen.

#### PVGIS software

PVGIS refers to geographic information systems (GIS) software designed specifically for PV systems or solar applications. PVGIS provides powerful capabilities for spatial analysis, site selection, and decision support in PV system deployment^29^. It can enhance the efficiency, accuracy and sustainability of solar projects by leveraging geospatial data and advanced modeling techniques^30^. PVGIS was used as a software program to collect all output data related to the PV system in order to predict the energy production of PV systems during the monitoring year, and the extent of the impact of changes in parameters such as system size, inclination angle, and module types, etc. PVGIS provides data that enables us to compare it with results obtained based on real data collected over the entire year. Solar radiation and temperature show the highest degree of variability and sensitivity among all factors affecting the performance of a PV system, which directly affects the final power output of the system^12^. Table 3 shows annual energy generation data for stationery and motor subfields, along with the corresponding average temperature and solar radiation (PVGIS 2016).

**Table 3:** Performance parameters results using solar PVGIS.

Year 2016	Temperature	Radiation	Fixed systems				Mtorised system		PV plant [energy]
Month	Temp [°C]	Ht-F [kWh/m²]	E_Mno-Si-F [kWh]	E_poly-Si-F [kWh]	E_A-Si-F [kWh]	E_Cd-Te-F [kWh]	E_Mono-Si-M [kWh]	E_poly-Si-M [kWh]	E_ PVGIS [MWh]
Jan	12.4	184.53	55 223.33	54 763.14	14 143.55	15 457.24	20 888.95	19 635.62	180.11
Feb	13.3	188.32	55 564.82	55 1 01.78	14 394.9	15 546.3	21 215.83	19 942.88	181.77
Mar	15.9	219.76	63 102.3	62 576.45	16 754.84	17 887.76	24 241.62	22 787.12	207.35
Apr	21.8	221.09	61 950.76	61 434.5	16 807.48	17 781.18	24 757.51	23 272.06	206.00
May	26.1	223.51	60 913.65	60 406.04	16 937.88	17 717.36	25 815.92	24 266.96	206.06
Jun	30.9	218.00	57 942.47	57 459.62	16 492.31	17 106.13	25 658.88	24 119.35	198.78
Jul	32.9	226.41	58 962.99	58 471.63	17 146.32	17 750.34	25 381.2	23 858.33	201.57
Aug	31.6	222.68	58 368.87	57 882.46	16 906.65	17 508.16	23 642.69	22 224.13	196.53
Sep	27.7	204.01	55 213.28	54 753.17	15 528.36	16 386.39	21 179.1	19 908.35	182.97
Oct	23.7	201.95	56 670.88	56 198.62	15 425.14	16 561.38	21 114.2	19 847.34	185.82
Nov	14.7	182.93	53 557.92	53 111.61	13 992.23	15 209.49	20 432.65	19 206.69	175.51
Dec	10.1	176.01	52 977.77	52 536.29	13 477.01	14 886.57	20 245.58	19 030.85	173.15

## Results and discussion

A monitoring period of 12 months, from January to December 2016, was used to evaluate the technical performance of the PV pilot plant using the proposed equations. The evaluation was conducted based on parameters specified in IEC 61724-1^43^, including final yield, reference yield, capacity factor, and performance ratio. The analysis compared actual measured values with simulation results obtained from the proposed equations.

### Annual measured values of radiation and temperature

**Fig. 4:**
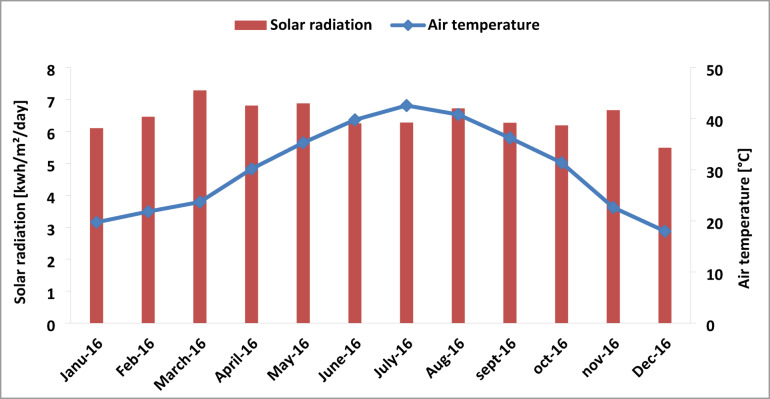
Monthly average daily of measured solar radiation and air temperature in oued Nechou Ghardaia.

To measure solar radiation and ambient temperature, a meteorological station equipped with a pyranometer was installed. Figure 4 illustrates the monthly evolution of average ambient temperature and average daily global incident radiation for the year 2016. Both climatic parameters varied throughout the year. The average temperature exceeded 42 °C in July during the summer season, while the lowest value, 16 °C, was recorded in December. Radiation levels also fluctuated throughout the year. As shown in the same figure, the highest radiation was recorded in March 2016 at over 7.1 kWh/m^2^/day, while the lowest was approximately 5.4 kWh/m^2^/day. Both temperature and radiation values are influenced by the seasonal climatic conditions.

### Simulated PVGIS results

**Fig. 5:**
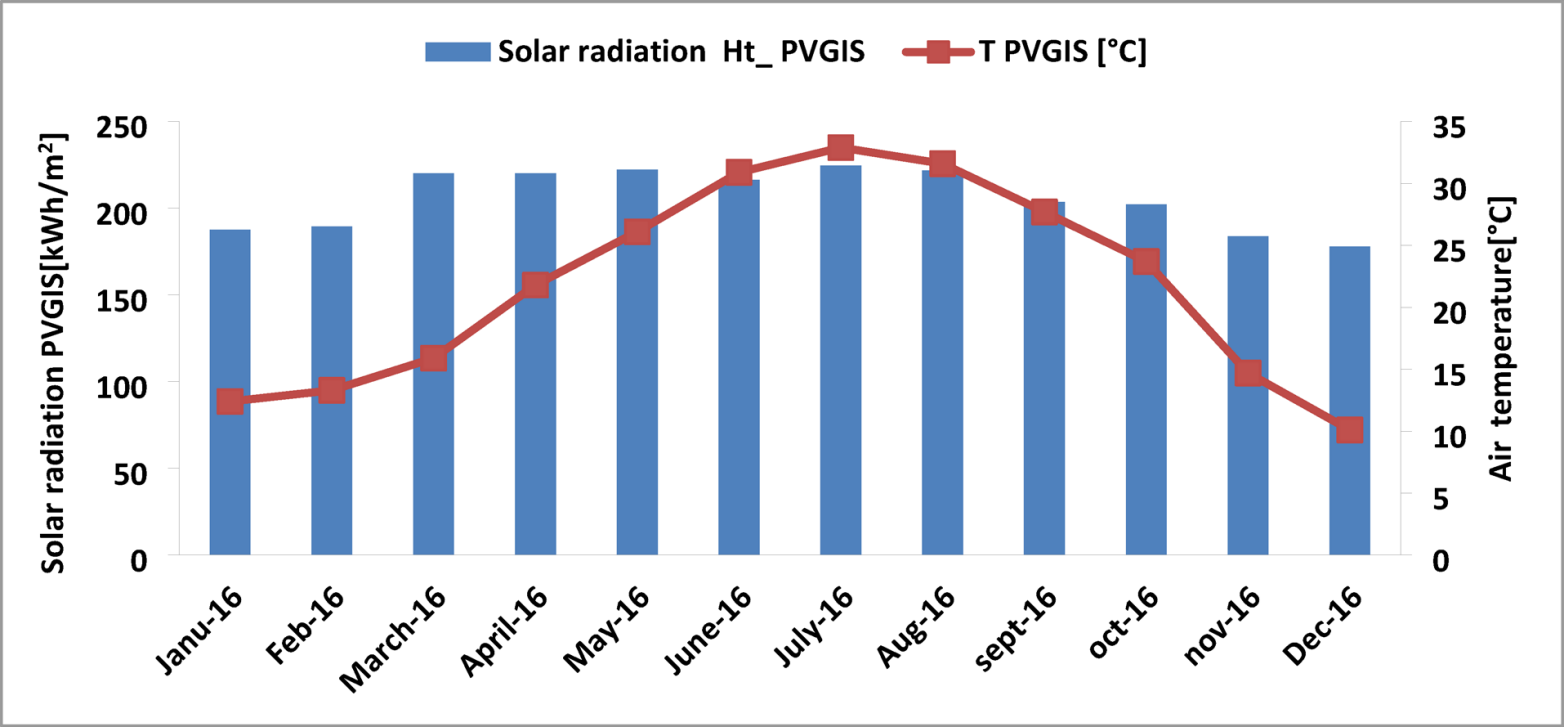
Monthly averages of PVGIS solar radiation and monthly air temperature in Oued Nechou Ghardaia.

Figure 5 illustrates the evolution of the monthly average ambient temperature as well as the monthly inclined global incident radiation for the year 2016. Both climatic parameters varied from one month to another^18^. The average temperature exceeds 43 °C in July, in the summer season; however, it is limited to the lowest value in December at 18 °C. The radiation throughout the year was different and in the same figure, the highest monthly solar radiation was recorded for July which was 225 kWh/m^2^, while the lowest was recorded in December, which was at 175 kWh/m^2^ . The temperatures and radiation depend on the climatic conditions of each season.

**Fig. 6:**
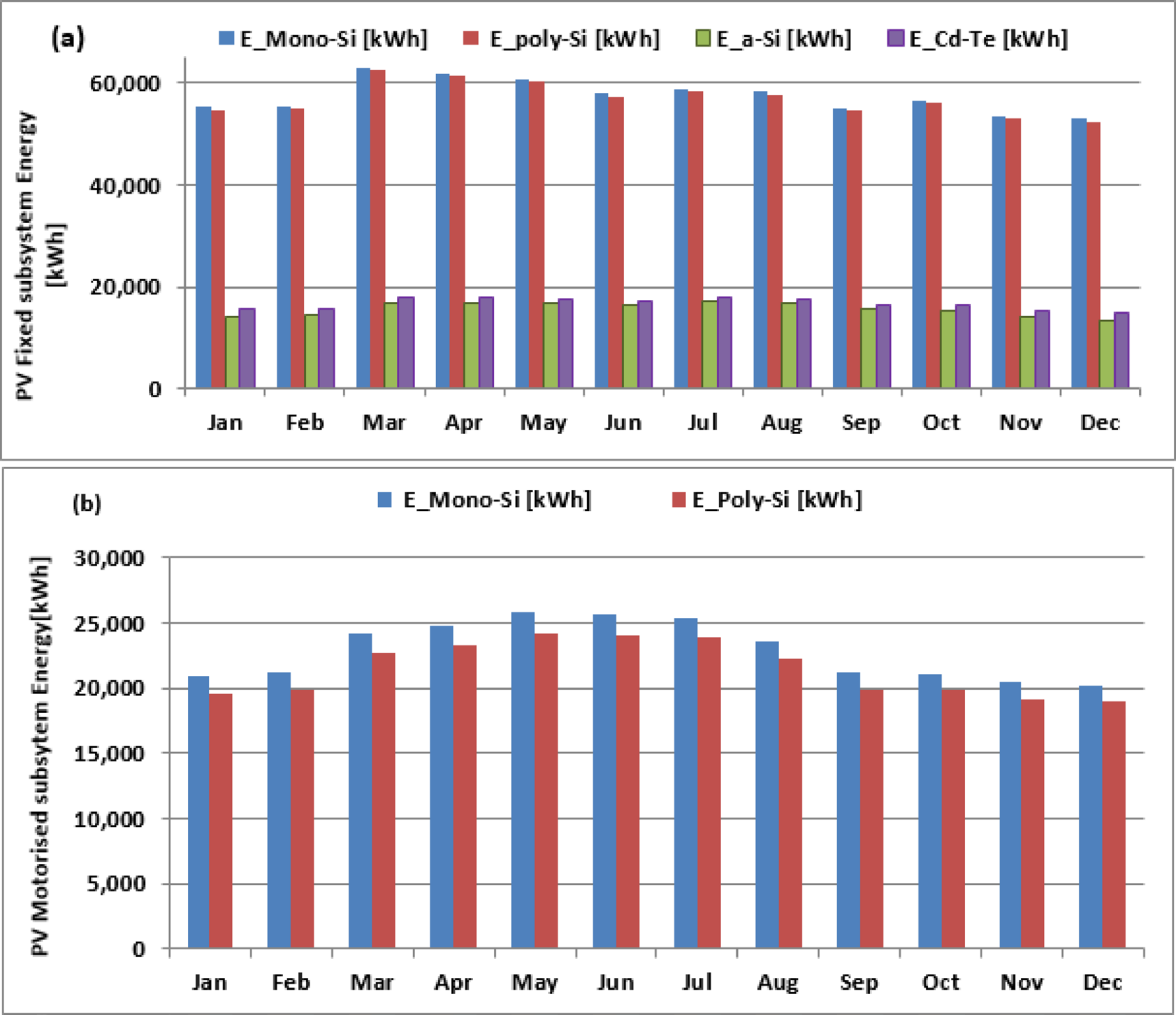
Simulated monthly average PV energy: (**a**) Fixed subsystem (Mono-Si, Poly-Si, a-Si, Cd-Te), (**b**) Motorized subsystem (Mono-Si, Poly-Si).

**Fig. 7:**
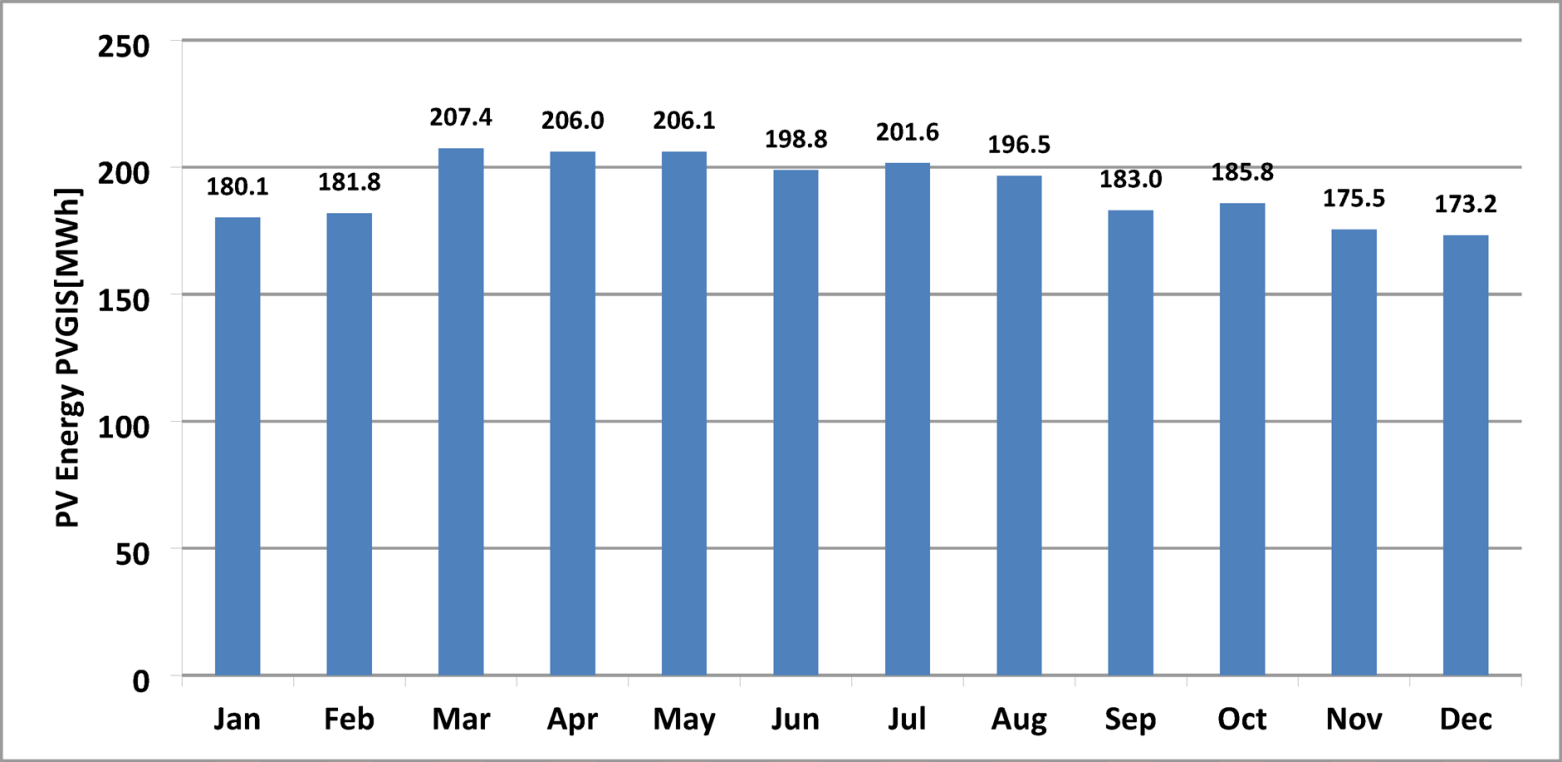
Total monthly average PV energy using PVGIS software.

Figure 6a displays the simulated energy generation from PVGIS for all fixed system technologies, while Fig. 6b presents the results for the motorized system. The combined energy output of all eight PV subfields for the year 2016 is illustrated in Fig. 7. The total energy production peaks during March, April, and May, with the monthly average surpassing 205 MWh.

### Comparison of simulation results (PVGIS) and measured data

**Fig. 8:**
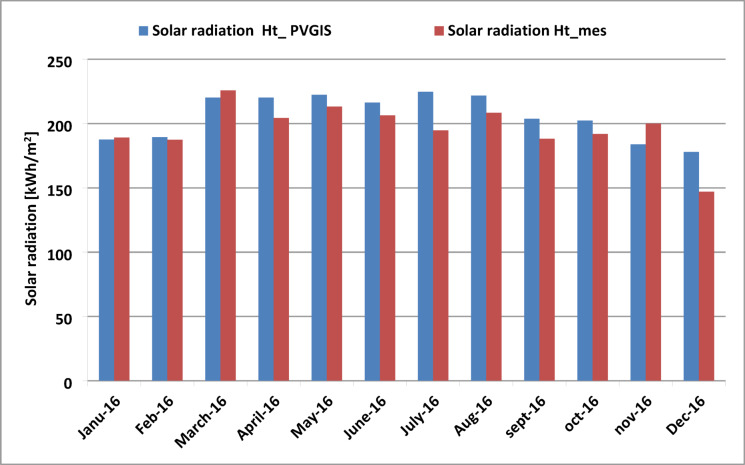
Comparison of monthly simulated solar radiation using PVGIS and measured solar radiation.

As shown in Fig. 8, the incident radiation reached its highest values in March and July 2016, both for measured data and for simulated data obtained using PVGIS. The lowest radiation value was recorded in December 2016. It is observed that the simulated values closely align with the measured ones for most months. However, the simulated values are slightly higher than the measured values throughout nearly the entire year. Climatic conditions such as temperature, irradiation, atmospheric pressure, humidity, and sandstorms significantly impact the performance of PV systems, particularly in harsh environments.

**Fig. 9:**
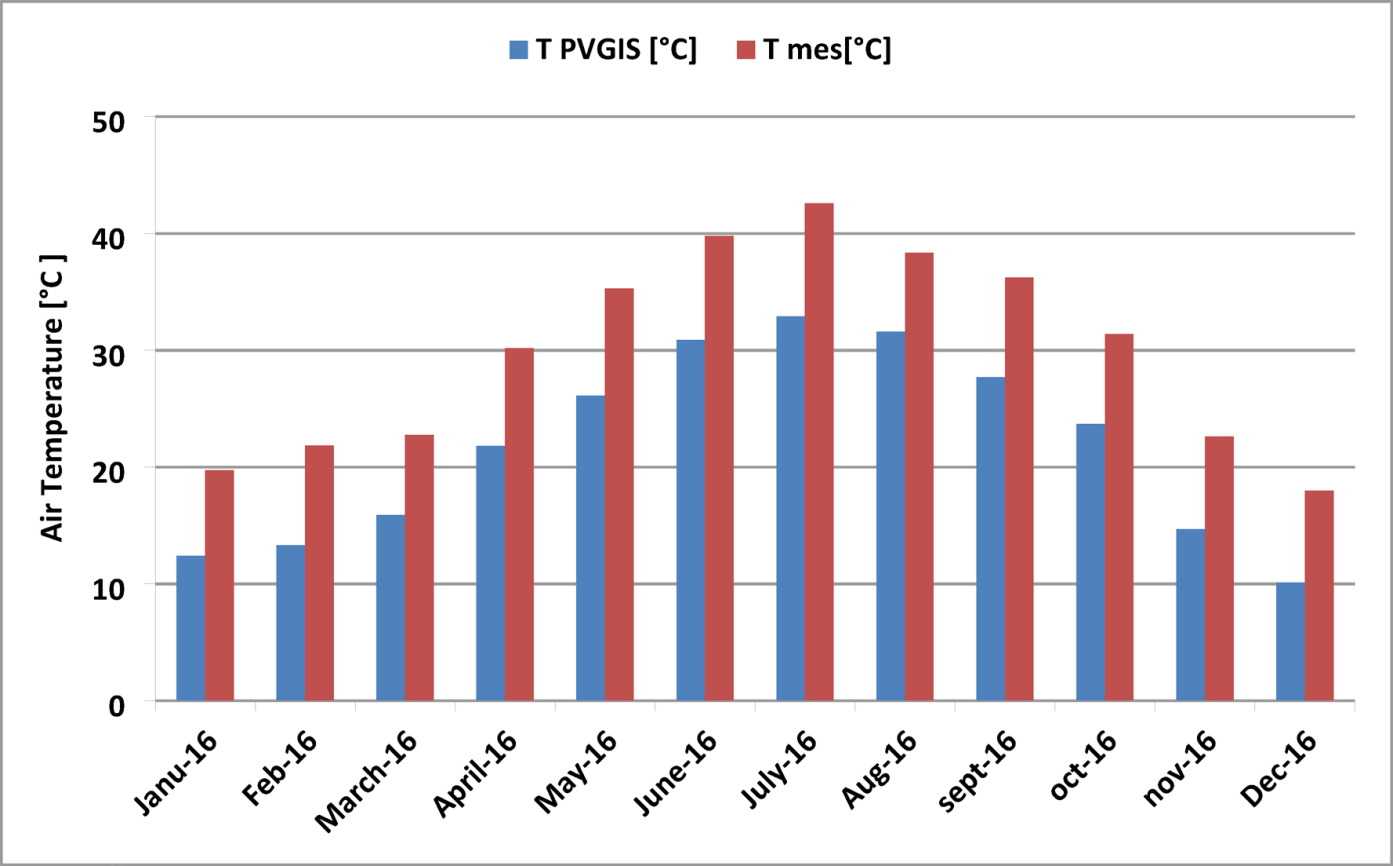
Comparison of simulated and measured average ambient temperature for the studied year 2016.

The simulated and measured average temperatures for each month of 2016 are presented in Fig. 9. It is observed that the shapes of the curves are very similar throughout the year, from January to December. However, the temperatures measured on-site are consistently higher than those simulated by PVGIS. The highest average temperature was recorded in July 2016, reaching 43 °C for the measured data and 33 °C for the PVGIS simulation. In contrast, the lowest average temperature occurred in December, with 18 °C measured and 10 °C simulated by PVGIS.

**Fig. 10:**
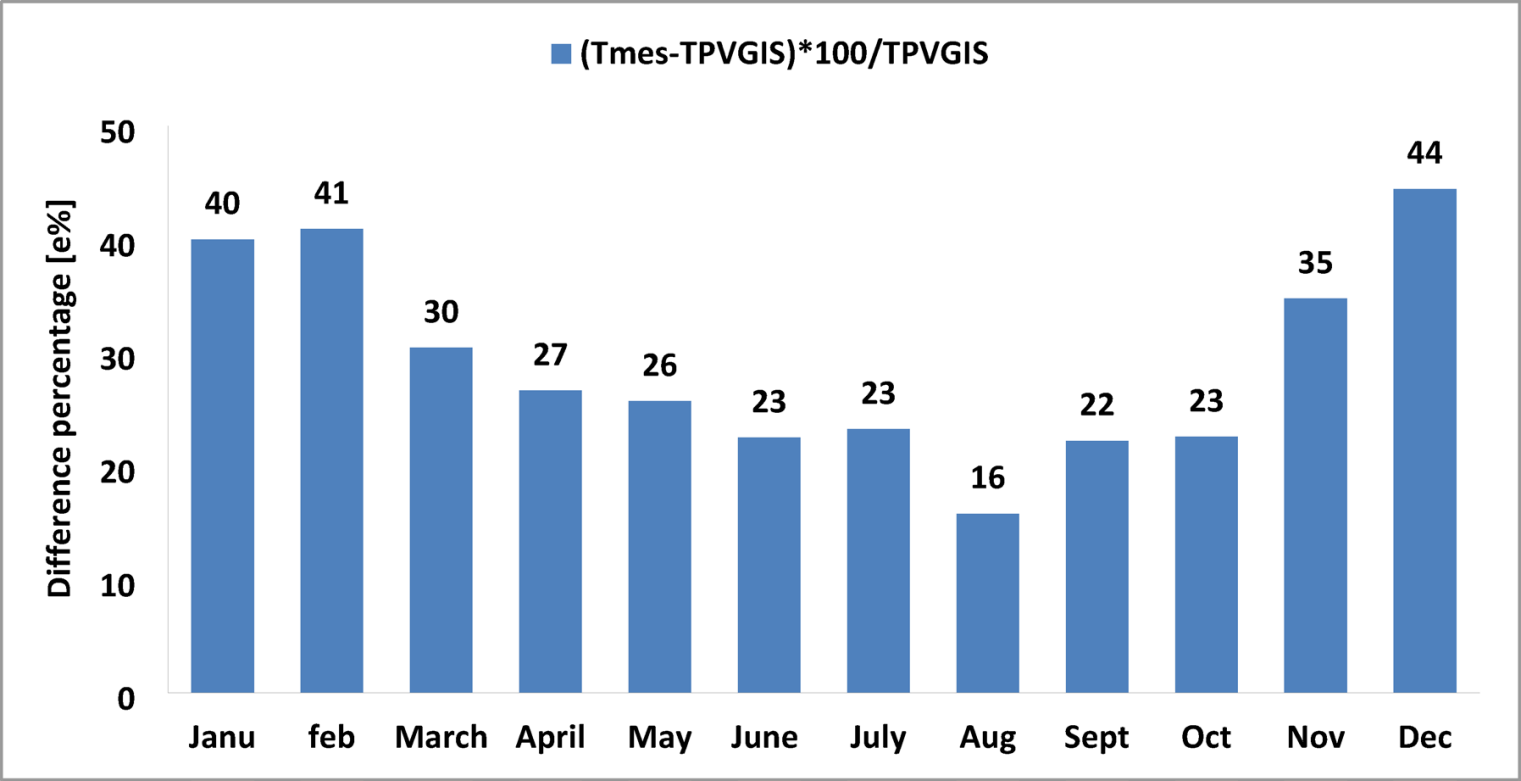
Percentage difference in temperature for the year 2016.

Figure 10 shows the percentage difference between the measured and simulated monthly average temperatures for the year 2016. It can be observed that during the three winter months (December, January, and February), there is a significant discrepancy between the measured and simulated temperatures, 44%, 40%, and 41%, respectively. In contrast, for the remaining months, the difference is generally lower, reaching less than 35%, with the smallest variation observed in August at 16%.

**Fig. 11:**
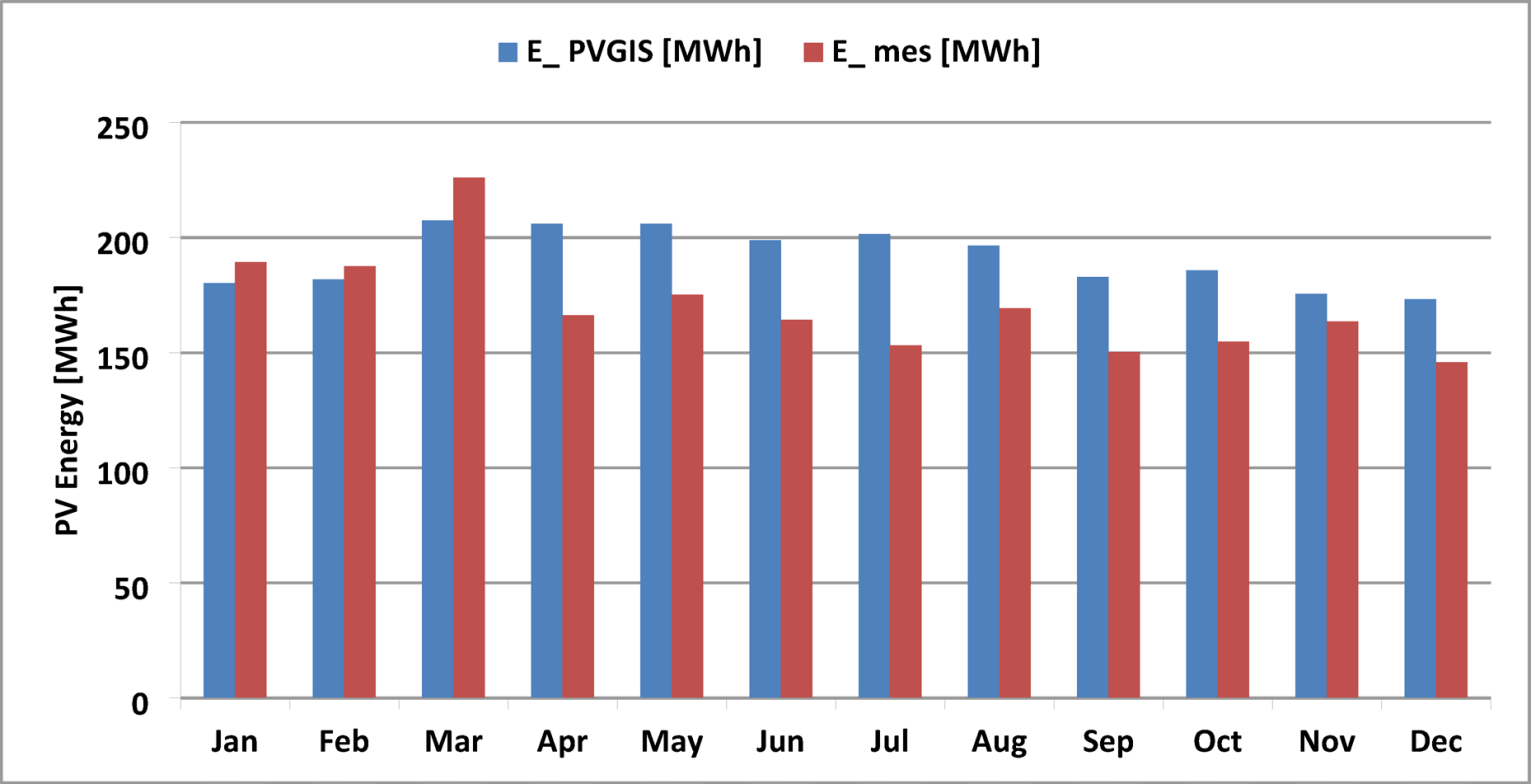
Comparison of monthly average simulated PV energy (E-PVGIS) and measured PV energy (E_mes) for the studied year 2016.

Figure 11 presents a comparison of the monthly average energy production measurements with the PVGIS simulation results. Climatic variables that can influence PV module temperature, and consequently affect outdoor performance, include ambient temperature, air mass, and the solar spectrum. The highest energy production (226 MWh) was recorded during the winter months (January, February, and March) of 2016, while production in the remaining months ranged between 145 MWh and 189 MWh. The PVGIS-simulated energy output for 2016 shows a similar monthly trend, although it overestimates production for three-quarters of the year. Notably, during the winter period (January–March), the actual output of the PV system was slightly lower than that predicted by PVGIS.

**Fig. 12:**
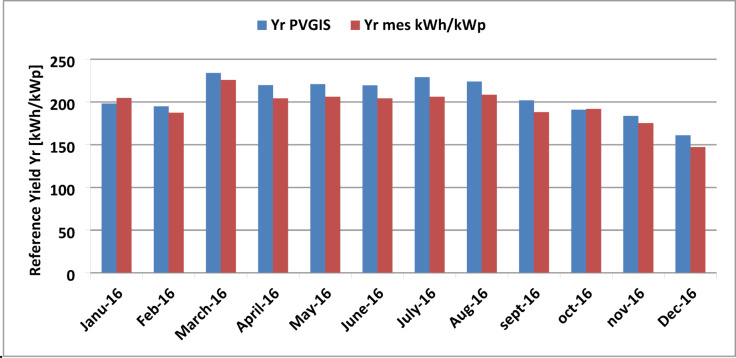
Comparison of simulated and measured monthly reference yields.

Figure 12 illustrates the reference yield, which represents the number of hours per month during which the solar radiation equals the reference radiation under standard test conditions (STC). Using performance factors, it becomes possible to compare PV systems with different configurations and technologies. The reference yield values were obtained from both PVGIS software and measured data recorded throughout the entire study year of 2016. Notably, the reference yield in October was the same for both PVGIS and measured data, approximately 191 h. In March, the reference yield reached its maximum, with 226 h for the measured value (Yr, mes) and 220 h for the simulated value. In July, PVGIS estimated 222 h, while the calculated value was 208 h. On the other hand, December showed the lowest reference yield, with 147 h measured and 187 h simulated.

**Fig. 13:**
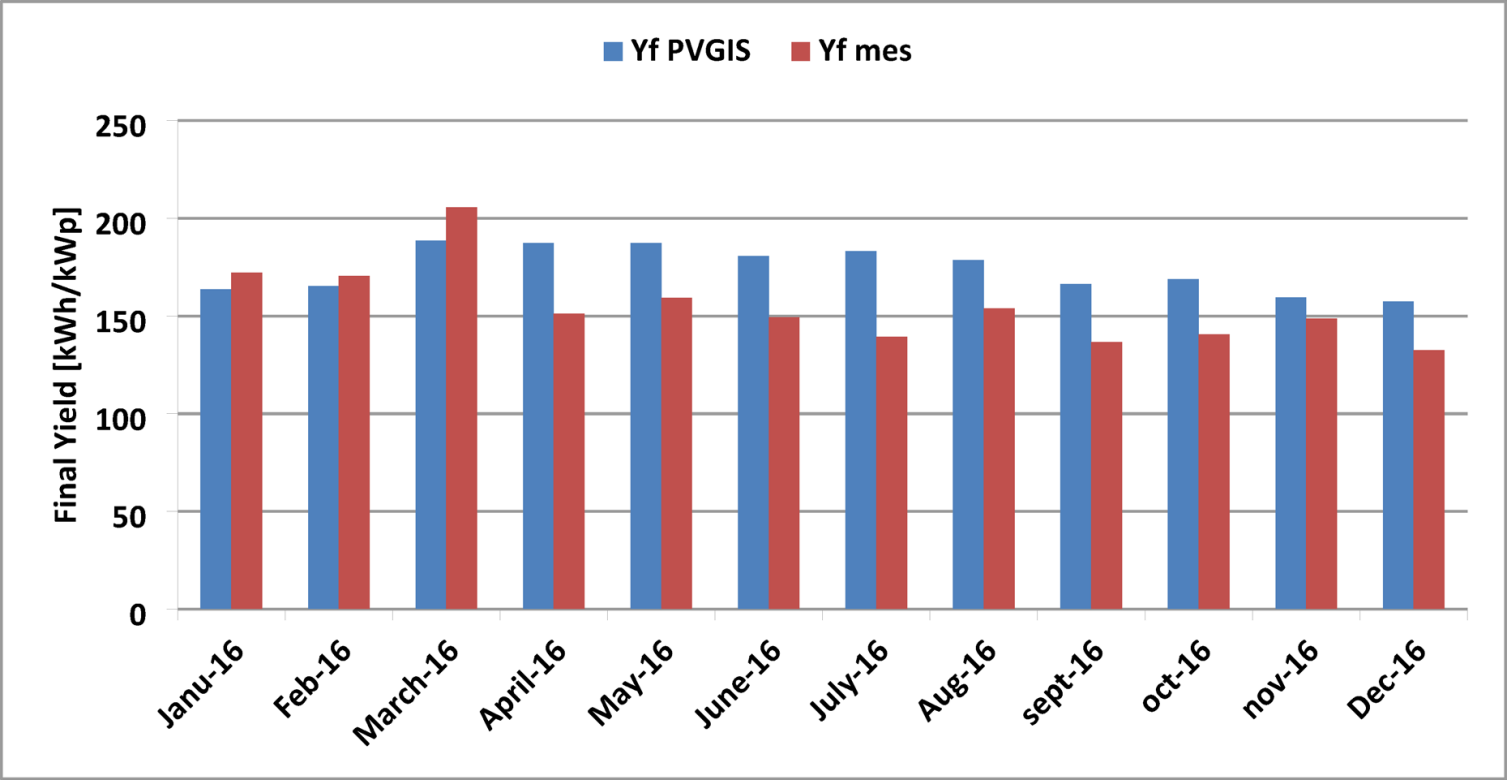
Comparison of simulated and measured monthly final yield.

The final yield (Yf) represents the number of hours per month that the plant operates at its rated capacity. Using the results obtained from the PVGIS program and the equations mentioned above, the Yf values can be calculated. For the measured values, Yf was highest at around 206 h in January, February, and March. However, it dropped to a minimum of 132 h in December. Similarly, the Yf value provided by PVGIS reached a maximum of 126 h in March and a minimum of 120 h in December. Figure 13 shows a comparison of the simulated and measured monthly final yield.

**Fig. 14:**
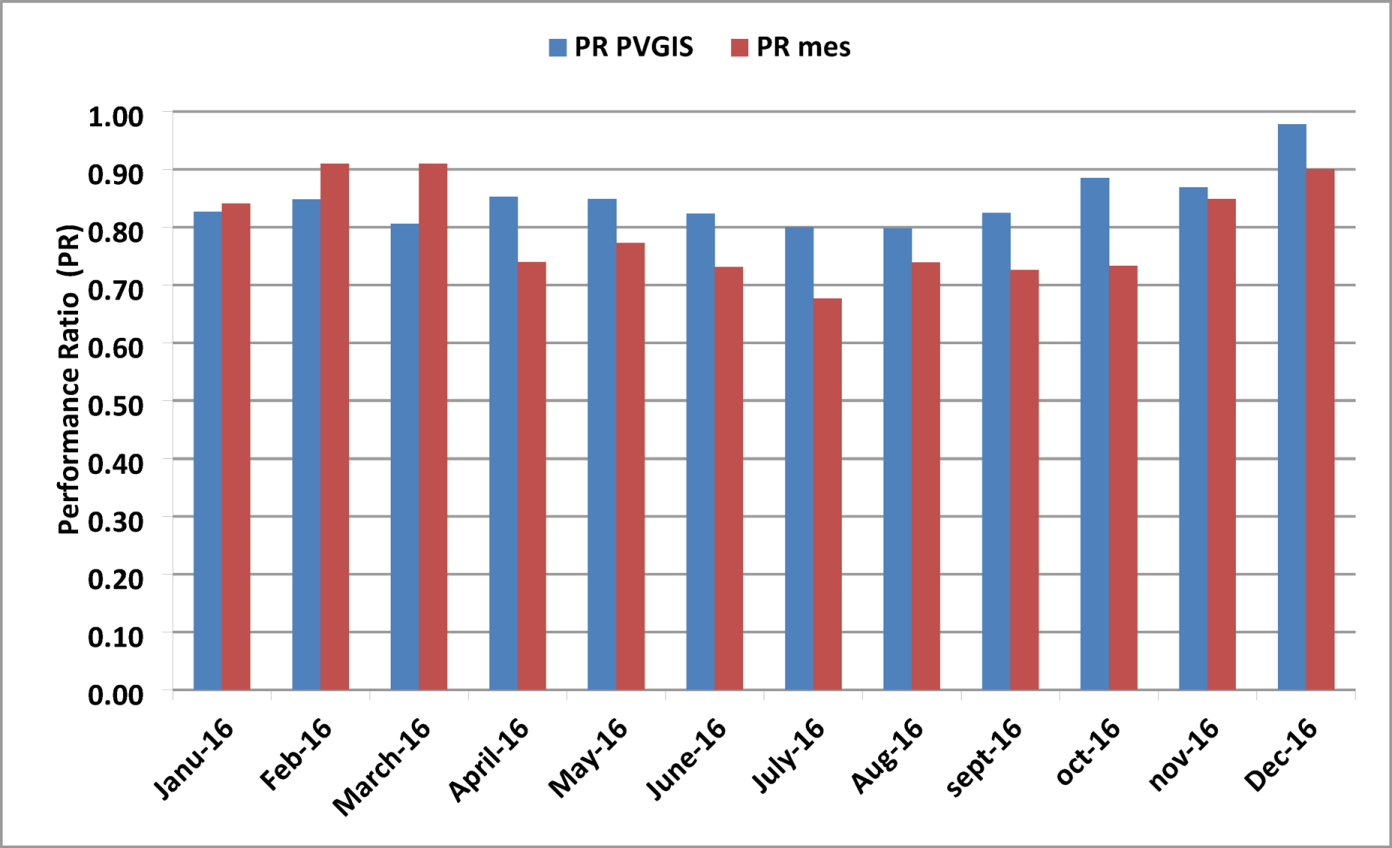
Comparison of simulated and measured monthly performance ratio.

Figure 14 presents the simulated and measured PR for the year 2016. It can be observed that the highest average measured PR values occurred in December, February, and March, reaching around 91%. In contrast, the average PR for the remaining months was approximately 72%, with November and January showing values around 85%. According to the PVGIS software, the highest simulated PR values were 98% in December, 89% in October, and 87% in November. The rest of the months yielded an average PR of approximately 82.5%. Overall, the measured PR values tend to be higher than the simulated ones, especially at peak levels.

**Fig. 15:**
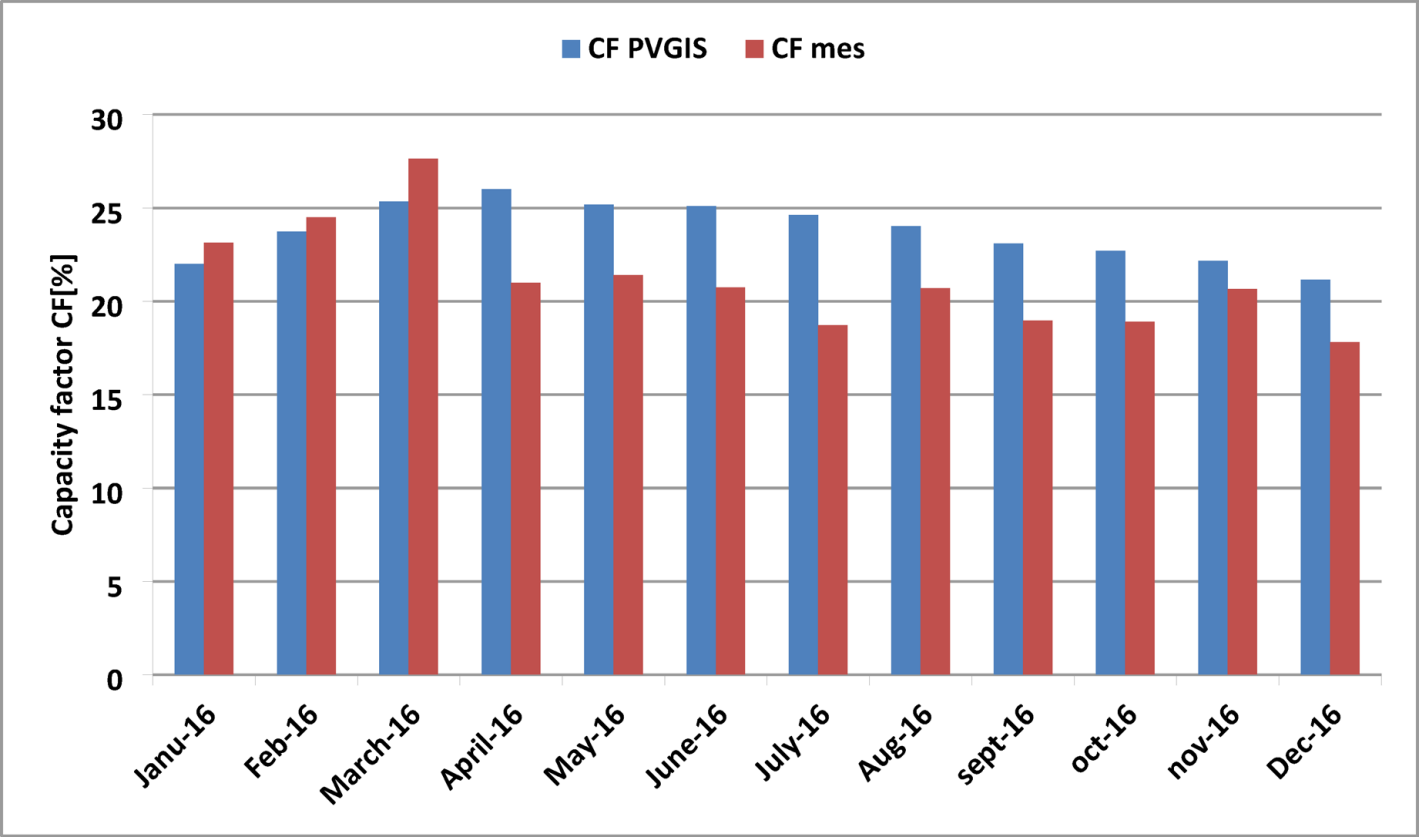
Comparison of simulated and measured monthly capacity factor for studied year 2016.

As shown in Fig. 15, the simulated and measured capacity factors are compared. The comparison indicates that January, February, and December were the periods during which both simulated and measured capacity factors reached their maximum values and were very close to each other for the same months. However, for the remainder of the year, the simulated values were consistently higher than the measured ones, with both following a similar trend.

**Fig. 16:**
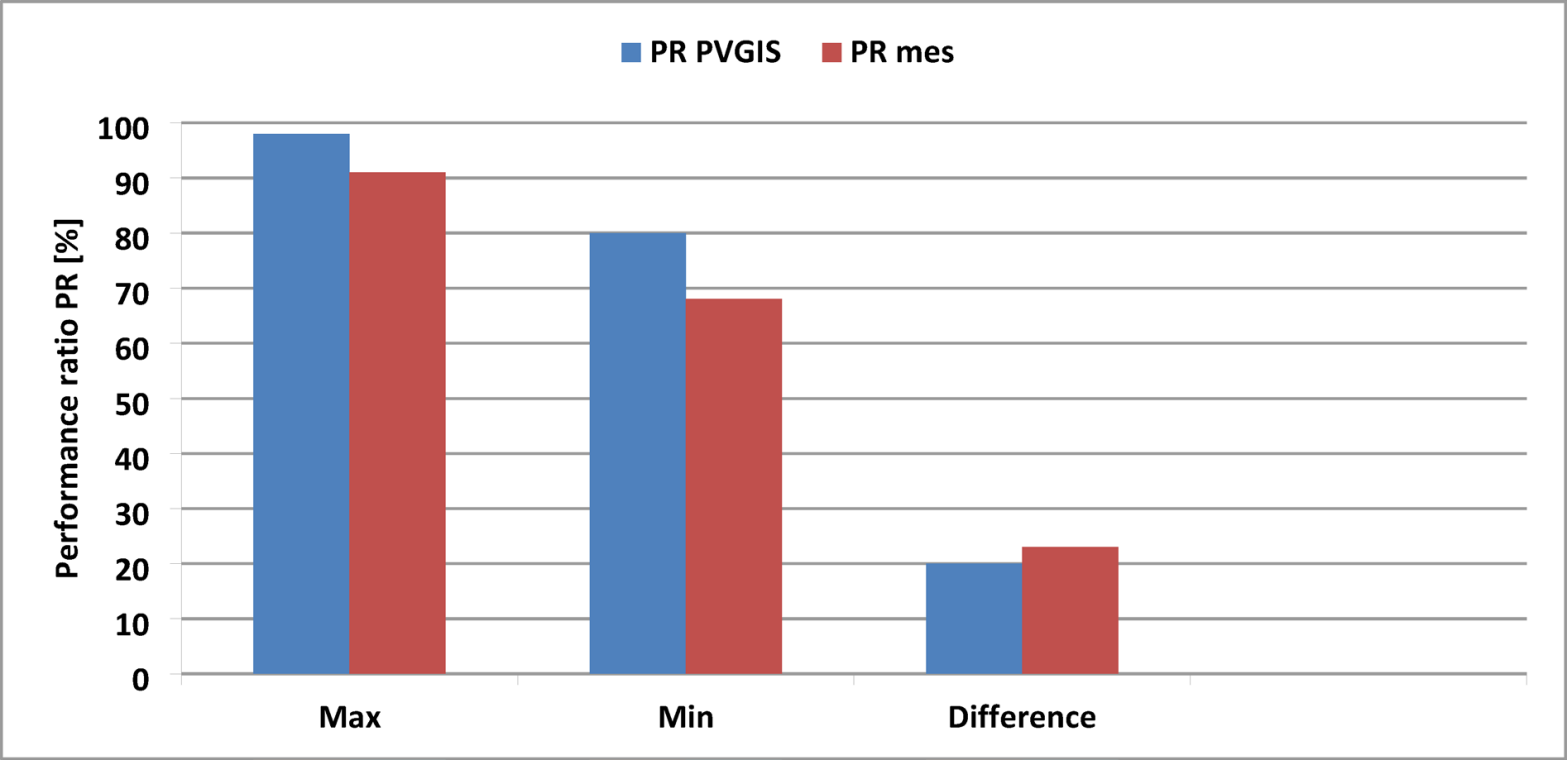
Comparison of simulated and measured (Max, Min, Difference) percentage performance ratio of the PV plant.

Figure 16 shows the minimum (Min), maximum (Max), and percentage difference in performance ratios of the PV system, which are closely aligned for both simulated and measured data throughout the study period. However, it is worth noting that the measured values exhibit slightly greater deviation compared to the simulated ones, particularly in the percentage difference, indicating some instability in certain meteorological parameters that affect the performance of the PV installation.

**Fig. 17:**
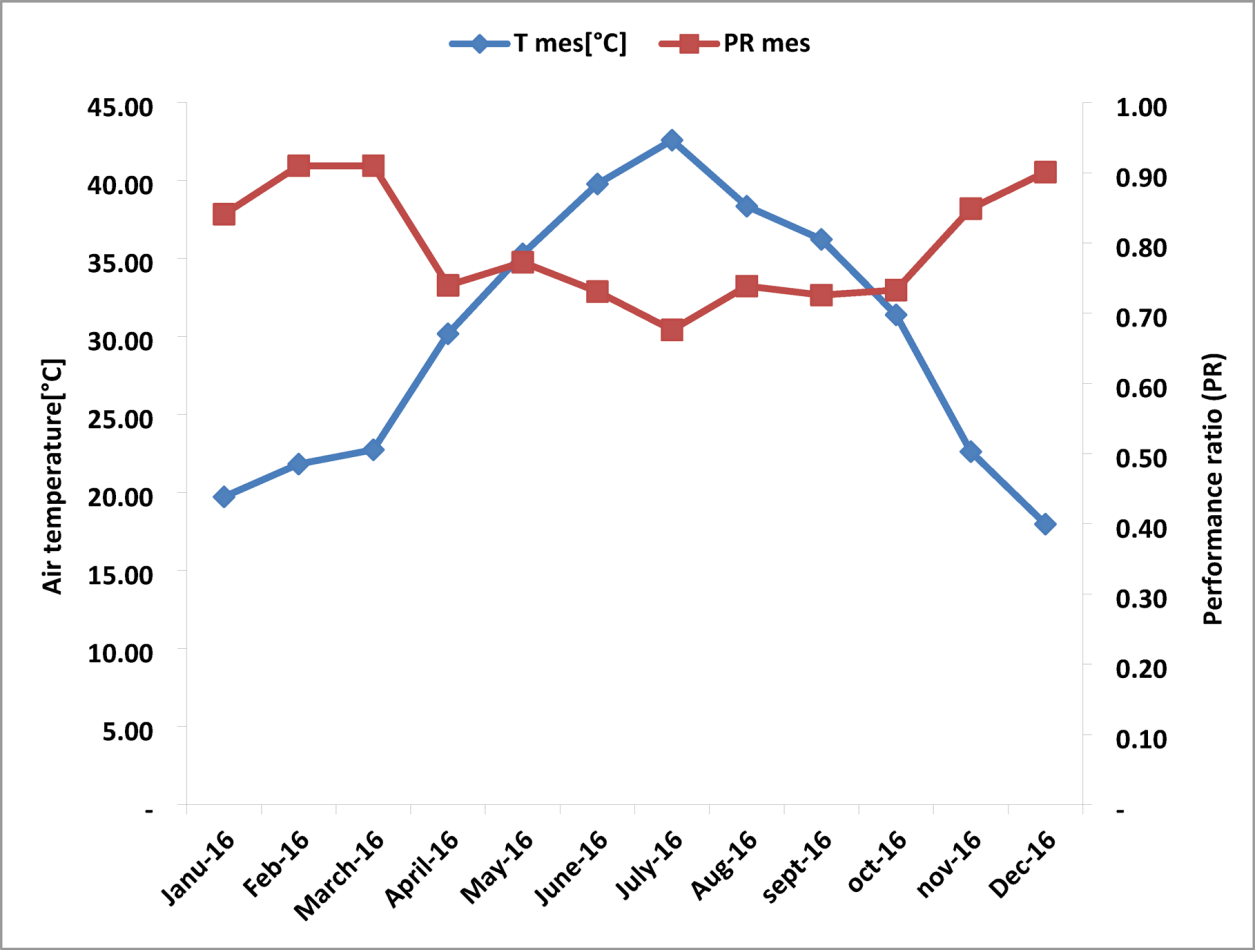
Monthly average ambient temperature and performance ratio for studied year.

Figure 17 shows the monthly PR and ambient temperature over the study period in 2016 for the PV system. The results reveal that the PR was highest at 0.91 in February, March, and December, while the lowest value, 0.68, was recorded in July. Clearly, the PR exhibits an inverse relationship with ambient temperature fluctuations.

**Fig. 18:**
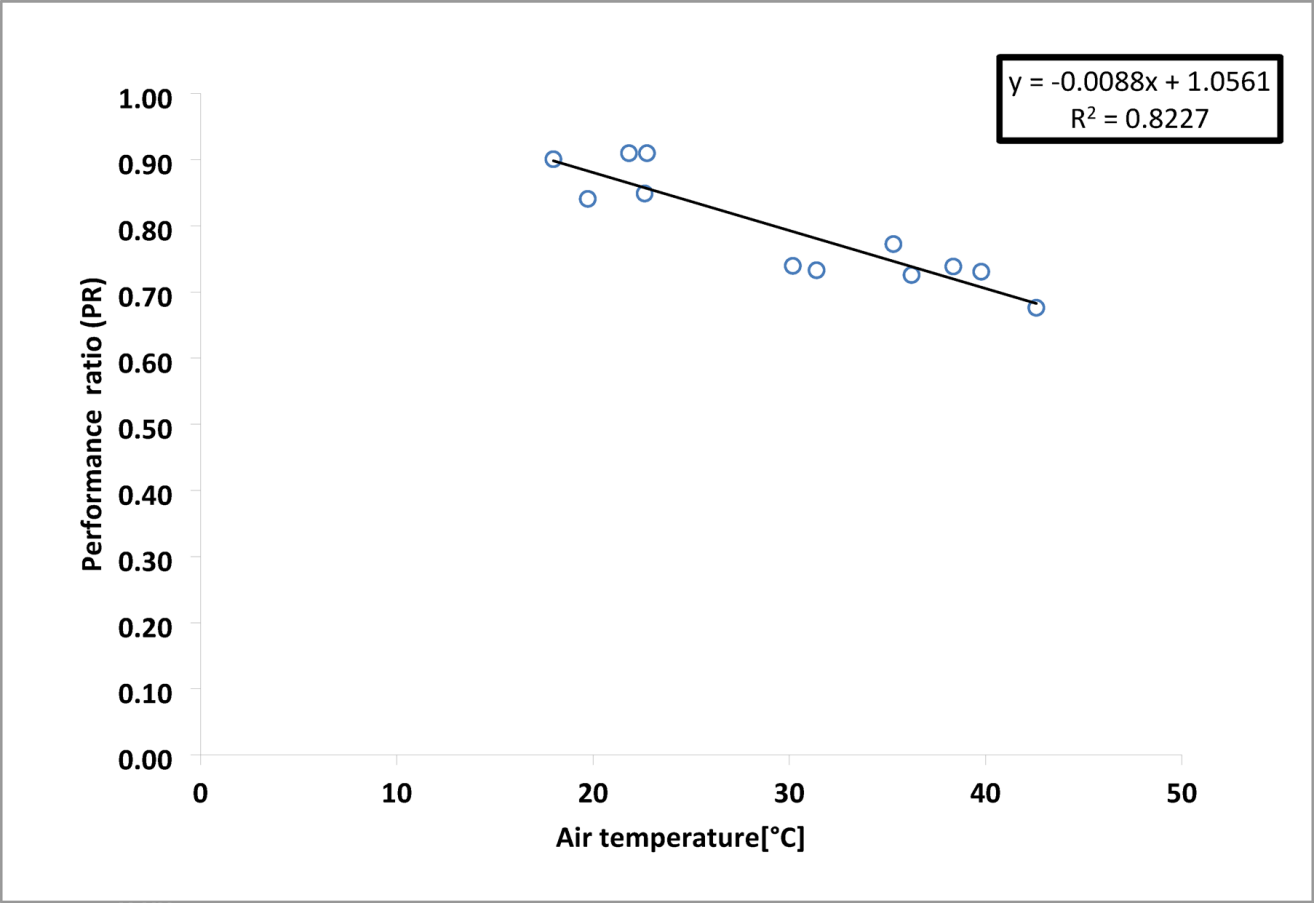
The correlation between performance ratio and ambient temperature.

Figure 18 reveals a negative correlation between temperature and the PR of the PV plant. The downward slope in the figure visually confirms that increasing air temperature reduces the PR, with a correlation coefficient (R²) of 0.8827.

8 $$\:\text{P}\text{R}=-\:0.0088\times\:{\text{T}}_{\text{a}}+1.0561$$

**Fig. 19:**
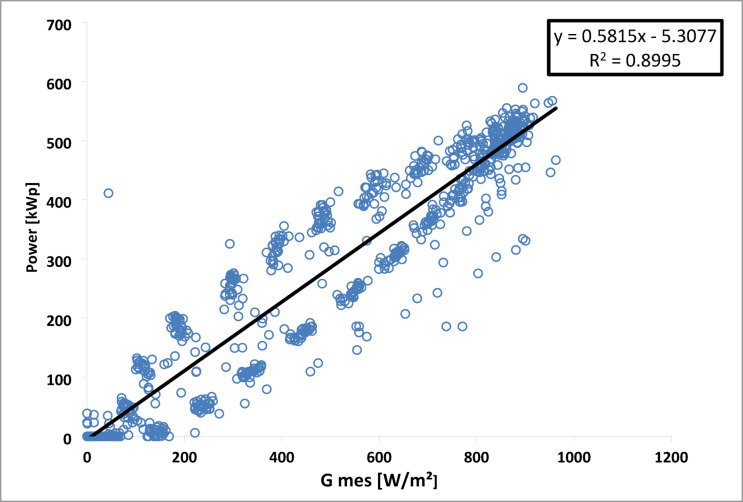
Variation of (P_ac_) as a function of irradiation for July 2016.

A linear progression of output power (P_ac_) with irradiation is observed, as shown in Fig. 19. The relationship between P_ac_ and irradiation (G) is defined below, with a correlation coefficient (R²) of 0.8995.

9 $$\:{\text{P}}_{\text{m}\text{e}\text{s},\text{i}}=-\text{5,3077}+\text{0,5815}{.\text{G}}_{\text{m}\text{e}\text{s},\text{i}}$$

**Fig. 20:**
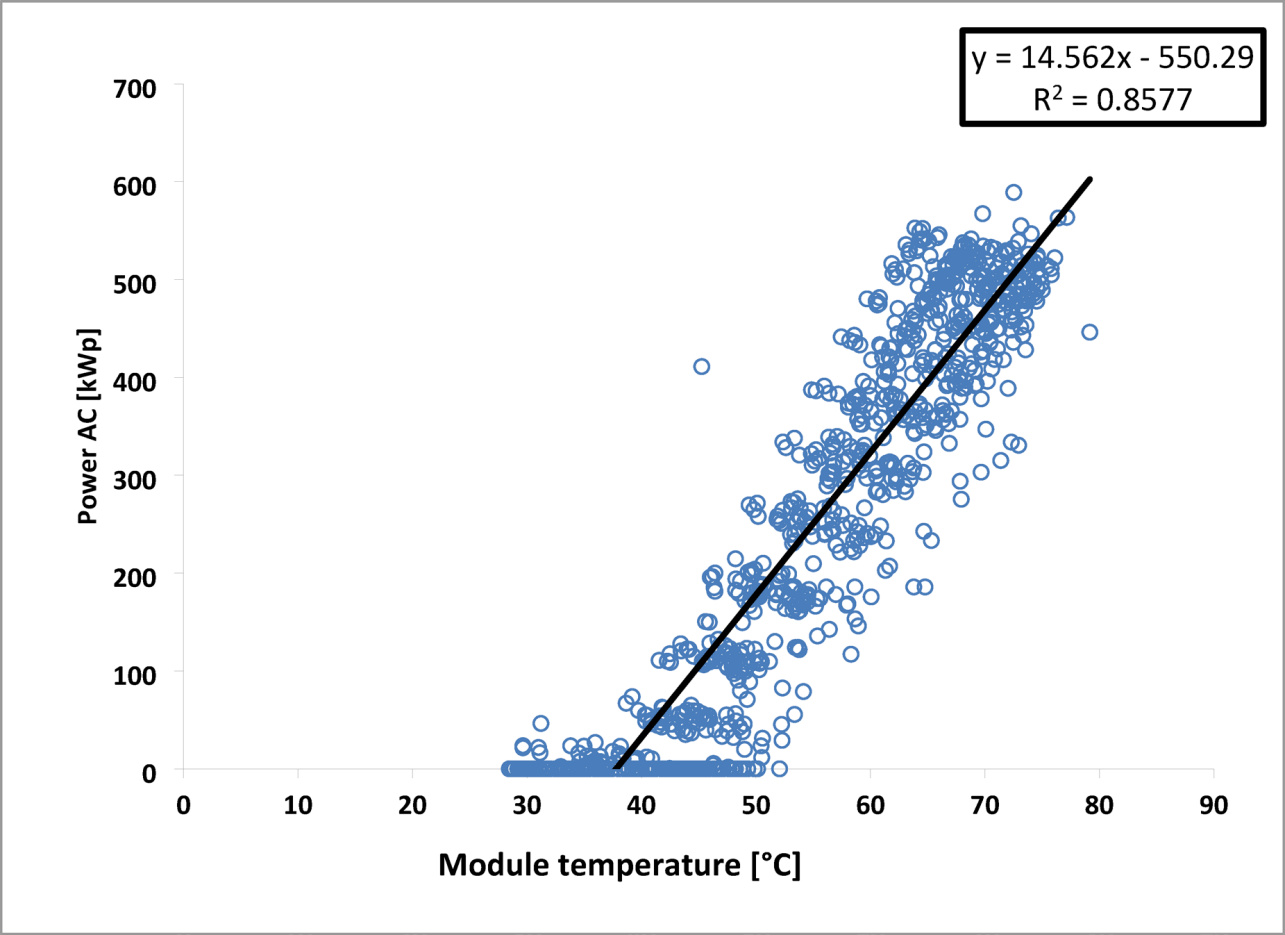
Variation of (P_ac_) as a function of Module temperature July 2016.

In the same way, another linear law relating the output power to the module temperature is shown in Fig. 20, with a correlation coefficient (R^2^) of 0.8577.

10 $$\:{\text{P}}_{\text{m}\text{e}\text{s},\text{i}}=-550.29+14.562{.\text{T}}_{\text{a}}$$

### Comparison of the grid-tied pilot PV plant with other PV plants around the world

A comparison of the performance indicators of the 1.1 MW_p_ PV pilot plant with those of other solar PV installations across Algeria and around the world is presented in Table 4. As shown in the table, the SPVP in Kuwait, which operates in a Saharan climate, has the highest performance ratio (84.5%), followed closely by the SPVPs in North India and Ghardaia, Algeria, each with 82% based on experimental results and 80% based on PVGIS simulations. The SPVPs in Malawi (monitored over four years) and Tangier, Morocco, both exhibit PR values of 79%, representing average performance under two distinct climatic conditions. In contrast, the lowest PR value (66%) is observed at the SPVP in Nouakchott, Mauritania, which also has a hot and arid climate. This study reveals superior performance ratios for the investigated PV pilot plant compared to installations in other locations, even those with similar environmental conditions. Several factors likely contribute to these performance differences, including the technology of the PV modules^43^, the inverter systems employed^44^, and the specific design configuration of each installation^7^. In summary, these findings suggest that Algeria offers favorable conditions for the efficient deployment of PV systems. The results underscore the country’s strong potential for harnessing solar energy and advancing its renewable energy goals.

**Table 4:** Performance ratio of PV plants compared with performance ratio of PV system reported elsewhere locations.

Location	Climate	Rated power	Performance ratio (%)	Monitoring period (year)	References
Adrar, Algeria	Saharan	20 MW_p_	71	1	^8^
Tangier, Morocco	Arid, Humid	5 kW_p_	79	1	^18^
Malawi	Cool dry	830 kWp	79	4	^28^
Jaddah, Saudi Arabia	Saharan	12.5 kW_p_	78	1	^14^
Nouakchott, Mauritania	Hot and arid	954.809 kW_p_	66	1	^33^
Northern India	Hot and arid	186 kW_p_	82	1	^24^
Kuwait	saharan	85.05	84.5	1	^31^
Ghardaia, Algeria	Saharan climate	1.1MW_p_	80	1	Present study

## Conclusion

This study evaluated the performance of PV plants under Saharan climate conditions, leading to the following key findings:

1. Performance Analysis: Eight PV subsystems, including two motorized, were analyzed using final output power (Eac) and PR to assess reliability and environmental impacts under harsh conditions.
2. PVGIS vs. Measurements: A comparison between PVGIS simulations and measured field data showed that PVGIS provides reliable estimates, with an annual mean PR of 80%, which can improve with motorized tracking across all subsystems.
3. Temperature Effects: An inverse relationship was observed between ambient temperature and PR. In cooler months (17–22 °C), PR averaged ~91%, while in hotter months (43 °C), it dropped to 68%.
4. Tracking System Benefits: Motorized tracking systems were shown to enhance energy yield, reduce shading losses, and improve system reliability under Saharan conditions.
5. Energy Correlation: A strong linear relationship was found between energy output and irradiation (R² = 0.899) and module temperature (R² = 0.857).
6. Design Implications: These findings have important implications for optimizing PV system design and operation in high-temperature, high-irradiance environments.
7. Pilot Plant Validation: The pilot plant is validated as a testbed for evaluating PV performance under extreme Saharan conditions, supporting its potential for broader deployment in similar regions.
8. Study Limitations: This study did not consider shading and windstorm effects, which should be explored in future research.
9. Overall Conclusion: The results demonstrate that motorized PV systems significantly improve energy production and operational stability in harsh desert climates.

## Data Availability

The data supporting the findings of this study can be accessed from corresponding author upon reasonable request.

## Abbreviations

A: Surface area m^2^
CF: Capacity factor %
E_AC_: Energy output (AC side) kWh
G: Solar irradiance (in-plane) W/m^2^
Gi: Incident irradiance W/m^2^
H: Total radiation kWh/m^2^
Isc: Short-circuit current A
NOCT: Nominal operating cell temperature °C
PR: Performance Ratio
R^2^: Coefficient of determination (correlation)
T_a_: Ambient temperature °C or K
T_c_: Module temperature °C or K
Tm: Mean temperature °C
T_NOCT_: Temperature at NOCT condition °C
Voc: Open-circuit voltage V
Vmp: Voltage at maximum power point V
Y_f_: Final yield kWh/kWp
Y_r_: Reference yield kWh/kWp
AC: Alternating current
CF: Capacity factor
DC: Direct current
GW: Gigawatt
IEC: International electrotechnical commission
MPPT: Maximum power point tracking
MW: Megawatt
MWp: Megawatt-peak
kWp: Kilowatt-peak
PR: Performance Ratio
PV: Photovoltaic
PVGIS: Photovoltaic geographical information system
SCADA: Supervisory control and data acquisition
SPVP: Solar photovoltaic plant
STC: Standard test conditions
